# Measurement of Oro-Cecal Transit Time in LPS-Treated Pigs Fed High and Low Fiber Diets Using the Lactose-^13^C-Ureide Test in Breath and Saliva Samples

**DOI:** 10.1021/acs.jafc.5c00534

**Published:** 2025-04-15

**Authors:** Mariagrazia Cavalleri, Quentin L. Sciascia, Solvig Görs, Andreas Vernunft, Henry Reyer, Klaus Wimmers, Jürgen Zentek, Jeannette Kluess, Sven Dänicke, Cornelia C. Metges

**Affiliations:** † Research Institute for Farm Animal Biology (FBN), 18196 Dummerstorf, Germany; ‡ Faculty of Agricultural and Environmental Sciences, University of Rostock, 18059 Rostock, Germany; § Institute of Animal Nutrition, Freie Universität Berlin, 14195 Berlin, Germany; ∥ Federal Research Institute for Animal Health, Institute of Animal Nutrition, 38116 Braunschweig, Germany

**Keywords:** oro-cecal transit time, dietary fiber, lipopolysaccharide, lactose-^13^C-ureide, gut, pig

## Abstract

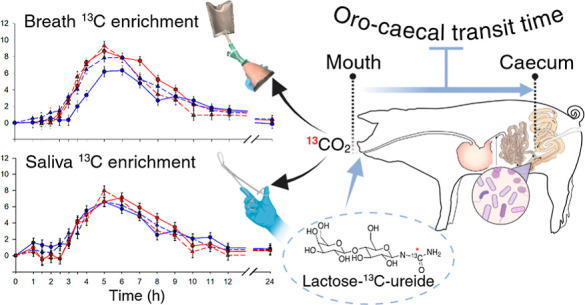

The lactose-^13^C-ureide (L^13^CU) test, used in humans to measure oro-cecal transit time (OCTT) in breath CO_2_, was assessed for its suitability in pigs as a noninvasive alternative to intestinal cannulation. The OCTT was determined with the L^13^CU test in breath and saliva samples when pigs were fed low or high dietary fiber (DF) (low fiber, 2.8% DF; high fiber 6.5% DF) diets, and 24 h after an *i.m.* injection with either lipopolysaccharide (LPS) or NaCl. The OCTT measured in breath was longer in LF-LPS than in LF-NaCl and HF-LPS groups (3.4 vs 2.9 h; *p* < 0.05). Additionally, LPS prolonged the OCTT of pigs, and DF prevented this effect. 90 % of OCTT estimates measured in saliva and breath CO_2_ did not differ. We conclude that the L^13^CU breath test is useful for determining OCTT in pigs; saliva is generally suitable as a sample matrix for OCTT, but its use requires further validation.

## Introduction

The gastrointestinal (GI) transit time is the time it takes for food to pass through the alimentary canal, where it is exposed to endogenous digestive enzymes and, further down the intestinal tract, to microbial enzymes. The upper GI tract (GIT) from mouth to the terminal ileum is where dietary nutrients are digested and absorbed, reflecting ileal or prececal digestibility. The methods used in pigs to determine transit time through the upper GIT include the slaughter technique,^1^ intestinal cannulation spanning various gut segments for direct sampling of intestinal content,^2^ together with the use of nondigestible liquid or solid phase markers,^3^ or gamma scintigraphy with radio-labeled markers.^4^ Due to increasing concerns about the welfare of experimental animals and the implementation of the 3R principles, there is a growing need for less invasive methods that can advance our understanding of porcine GI function. The hydrogen breath test utilizing lactulose has been widely used as a noninvasive technique to measure oro-cecal transit time (OCTT),^5^ but lactulose osmotically accelerates intestinal transit.^6^ In contrast, the stable isotope-labeled substrate lactose-^13^C-ureide (L^13^CU) does not affect the motility of the small intestine (SI) and has been validated as a noninvasive marker for OCTT in clinical settings.^9,10^ Lactose-ureide (LU) is a glycosyl-ureide that cannot be metabolized by the enzymes in the SI in humans and reaches the large intestine (LI) intact^10^ or as glucose-^13^C-ureide (G^13^CU), since the brush border enzymes can hydrolyse the disaccharide bond in LU to glucose-ureide and galactose. A fraction of G^13^CU appears in the urine unchanged.^7,8^ Only the bacterial enzymes of *Clostridium innocuum*, present in the human large intestinal microbiome, can split the sugar-ureide bond.^11^ However, to date, it is not known whether enzymes of other intestinal bacteria can do so. The breakdown of the sugar-ureide bond leads to the release of ^13^C-urea, which is metabolized by urease into ammonia and ^13^CO_2_, subsequently exhaled with breath.^5^ By tracking the enrichment of breath ^13^CO_2_, an indirect measure of OCTT can be obtained. In addition to clinical use in humans,^9,10,12^ the L^13^CU breath test has been used in horses^13^ and rats,^14^ but the L^13^CU breath test has never been used to measure OCTT in pigs. Additionally, the noninvasive nature of saliva collection makes it an interesting biological sample matrix to assess health and investigate potential biomarkers of inflammation.^15^ However, saliva has never been employed for monitoring OCTT; hence, this investigation could contribute to its wider use in the future.

Dietary fiber (DF) is one of the many factors that can influence the digesta passage time through the GIT^16^ due to its physicochemical properties^17^ or its microbial fermentation products, the short chain fatty acids (SCFAs) like butyrate,^18^ which can regulate GI motility.^19^ Also, lipopolysaccharide (LPS), a well-characterized immune system challenge in pigs, has been reported to affect the transport of nutrients, compromise the normal GI functionality, as well as influence the GI motility.^19−21^ DF can protect the gut against pathogens,^22^ and prebiotics like galacto-oligosaccharides, altering gut microbiota composition,^18^ were found to alleviate LPS-induced effects.^23^ Therefore, the primary aim of this study was to use L^13^CU for the first time in pigs to measure ^13^CO_2_ abundance kinetics in breath and to calculate OCTT. Second, the OCTT was investigated with the L^13^CU breath test to examine the effects of two diets containing different amounts and types of fiber and 24 h after administration of LPS. The aim was to determine whether the motility of the digestive tract returns to a physiological state 24 h after injection of LPS and whether DF could support this. Third, saliva was used to measure OCTT to determine whether saliva could replace breath as a less invasive sample probe. Finally, we investigated whether *C. innocuum*, the responsible bacterium for the LU bond cleavage in human studies, could be detected in the feces of the pigs used.

## Materials and Methods

### Animals and Diets

All experimental procedures were performed according to the guidelines of the German Animal Welfare Act in compliance with Directive 2010/63/EU and were approved by the State Office for Agriculture, Food Safety and Fishery, Mecklenburg-Western Pomerania, Germany (LALLF M-V/TSD/7221.3-1-039/21).

14 German Landrace sows (parity 2–9) bred at the Research Institute for Farm Animal Biology (FBN), Dummerstorf, Germany, were fed standard pregnancy and lactation diets^24^ (Table S1). Two to four male piglets per litter (average birth weight 1.58 ± 0.25 kg) were preselected for the study immediately after farrowing. The study was conducted across 8 experimental runs with 44 experimental piglets in total. Creep feed (Table S1) was offered to the piglets from age day (d) 14. At d 28, pigs were weaned and fed a standard weaning diet until d 35 and switched to a pig starter diet (Table S1) until they were transitioned to the experimental diets at d50. At d 42, siblings with similar bodyweight (BW) (mean BW 12.0 ± 0.34 kg) were transferred to the experimental stable, where 4–6 selected pigs were group-housed in 6.7 m^2^ pens with a slatted floor, partly covered with rubber mats. Room temperature and humidity were kept at 22 °C and 40–60%, respectively. Lights were on from 7:00 to 19:30 h. At d 50, littermate pairs of similar BW (HF: 14.9 ± 0.33 kg; LF: 15.1 ± 0.34 kg; *p* > 0.1) were randomly assigned to either a high (HF) (*n* = 22) or a low fiber (LF) (*n* = 22) diet. The HF and LF diets were based on the same basal diet (Table 1), to which either 4.5% of lignocellulose plus 6.2% sugar beet pulp (HF) or 14.5% wheat starch (LF) was added and produced in meal form by the Institute of Animal Nutrition at the Federal Research Institute of Animal Health (Friedrich Löffler Institute, Braunschweig, Germany). Nutrient analysis (Table 1) was performed by Landwirtschaftliche Untersuchungs- and Forschungsanstalt der LMS Agrarberatung GmbH, Rostock, Germany, and a detailed fiber analysis was conducted at Professor Bach Knudsen’s lab at Aarhus University^25^ showing 2.3 times more cellulose and a somewhat higher proportion of soluble nonstarch polysaccharides (NSP) (29%) in the HF than in the LF diet (23%) (Table 2).

**Table 1:** Ingredient Composition and Analyzed Nutrient Content of Low and High Fibre Diets

items	low fiber	high fiber
ingredient composition, g/100 g fresh matter		
rye	17.4	17.4
wheat	18.6	18.6
corn	20.0	20.0
soybean meal 42	25.2	25.2
mineral premix^a^	2.64	2.64
l-lysine-HCl	0.31	0.31
dl-methionine	0.11	0.11
l-threonine	0.10	0.10
acid-insoluble ash^b^	1.00	1.00
soybean oil	0.20	4.00
wheat starch^c^	14.5	
lignocellulose (Arbocel)^d^		4.50
sugar beet pulp		6.20
analyzed nutrient content, g/kg fresh matter		
dry matter	890	896
ash	57.5	60.0
crude protein	173	175
crude fat	22.0	58.5
crude fiber	24.5	56.0
ADF om	30.0	71.5
aNDF om	83.5	129
ADL	4.50	16.5
starch	477	374
total sugar (saccharose)	44.8	44.0
calcium	8.70	8.85
phosphorus	5.45	5.25
sodium	1.80	1.65
magnesium	1.75	1.75
potassium	9.00	8.70
metabolizable energy [MJ/kg]	13.7	13.1

a Vaumin Mast 14, no. 43066; Vilofoss Neuenkirchen/Germany, provided per kg of premix: Ca 250 g, P 60 g, Na 55 g, Mg 10 g, vitamin A 2,16,000 IU, vitamin D3 40,000 IU, vitamin E 1200 mg, iron (sulfate monohydrate) 4000 mg, copper (sulfate pentahydrate) 500 mg, Mg (oxide) 2670 mg, Zn (oxide) 2500 mg, I (Ca iodate) 67 mg, sodium Se 13.5 mg.

b >97% SiO_2_; Sipernat22S, Evonik Industries, Hanau-Wolfgang/Germany.

c Native wheat starch HAMSTARCH A, Jäckering Mühlen- and Nährmittelwerke GmbH, Hamm, Germany.

d Arbocel RC fine, J. Rettenmaier & Söhne GmbH & Co KG, Rosenberg, Germany.

**Table 2:** Analyzed Composition of Carbohydrates and Fibrous Ingredients of the Low and High Fibre Diets^a^

items^b^	low fiber			high fiber		
Carbohydrates						
beta glucan	0.52			0.47		
Glucose	1.49			1.24		
Fructose	0.17			0.26		
Sucrose	3.56			3.53		
Fructan	0.72			0.79		
nonstarch polysaccharide	S-NSP	I-NSP	T-NSP	S-NSP	I-NSP	T-NSP
Rhamnose	0.04	0.04	0.08	0.10	0.05	0.15
Fucose	0.03	0.04	0.08	0.05	0.04	0.09
Arabinose	0.80	1.63	2.43	1.39	1.83	3.22
Xylose	0.52	2.14	2.66	0.48	2.36	2.84
Mannose	0.10	0.40	0.50	0.13	0.83	0.96
Galactose	0.72	0.75	1.47	1.04	0.79	1.83
Glucose		1.10	1.08	0.53	0.78	1.31
uronic acid	0.42	0.57	0.98	1.37	0.65	2.02
Cellulose		2.13	2.13		4.99	4.99
NSP	2.61	8.79	11.40	5.09	12.31	17.40
Lignin		0.99	0.99		2.04	2.04
dietary fiber	2.61	9.79	12.39	5.09	14.35	19.44

a Abbreviations: NSP, nonstarch polysaccharides; S-NSP, soluble NSP; I-NSP, insoluble NSP; T-NSP, total NSP.

b As % of dry matter (DM) content: LF % DM = 88.7; HF % DM = 88.2.

**Figure 1:**
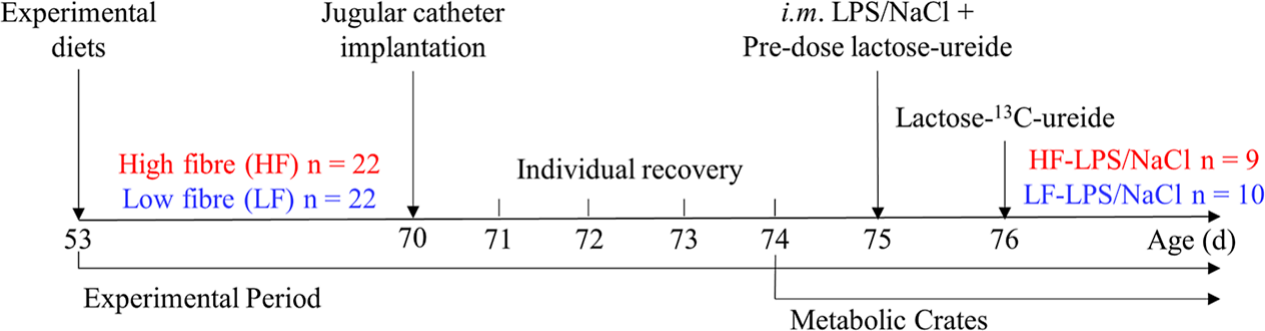
Schematic representation of the experimental procedure. Pigs were fed either low (LF, *n* = 22) or high fiber (HF) (*n* = 22) diets. At age d 70, a jugular catheter was implanted, and after 3 days of recovery, pigs were transferred into metabolic crates. At d 75, half of the pigs in each group received an *i.m.* LPS injection leading to four subgroups (LF-LPS, LF-NaCl, HF-LPS, and HF-NaCl), and all pigs received a predose of unlabeled lactose-ureide to stimulate the bacteria responsible of cleaving its bond. At d 76, the lactose-^13^C-ureide test was performed.

Piglets were transitioned to the experimental diets over 3 days, with 25, 50, and 75% of the respective experimental diet replacing their nursery diet, until 100% was consumed at the start of the experiment at d 53 (Figure 1). The HF and LF diets were fed iso-energetically twice daily (07:30 h, 16:30 h) based on the recommended daily energy and feed intake for pigs with a daily BW gain of 500 g between 8 and 11 weeks of age.^24^ Drinking water was provided ad libitum. At d 70, jugular vein catheters were fitted as described^26^ for repeated blood sampling in the LPS study (immunological results to be reported elsewhere). At d 74, pigs were weighed (mean BW: HF 28.1 ± 0.5; LF 28.6 ± 0.51 kg; *p* > 0.1) and transferred to metabolic crates (0.72 m^2^).

### Experimental Design

At d 75, 24 h before the administration of L^13^CU, half of the pigs per dietary group received a treatment (Trt) with an *i.m*. LPS injection (30 μg/kg BW; LPS from *Escherichia coli* O111:B4; SIGMA L2630), while the other half was injected with saline solution (NaCl) as a control. In the evening, 12 h after the injection and 12 h prior to the L^13^CU administration, 5 g of the respective experimental feed mixed with water (4 g) plus 400 mg of unlabeled LU (Campro Scientific GmbH, Berlin, Germany) powder were fed by hand to all the pigs to ensure they ate it entirely. This predose was administered to stimulate the enzyme activity of the large intestinal bacteria responsible for cleaving the LU bond.^10,27^ Feed was then withdrawn overnight (12 h), and at d 76 (24 h post-LPS challenge), all pigs were hand-fed feed portions containing 400 mg of L^13^CU powder^9^ (99 atom % ^13^C; Campro Scientific GmbH). The dosages of LU and L^13^CU and administration times were based on our pretrial results (Table S2 and Figure S1) and published data.^27^ Immediately after the pigs had consumed the L^13^CU-labeled feed, they were fed 33% of the daily amount of their respective experimental diet. The feed allowance on the L^13^CU test day was 1.26 kg in the LF group and 1.33 kg in the HF group (fresh matter). Two subsequent meals (33%) were given 4 and 10 h after L^13^CU administration. Out of the 44 experimental pigs, six were excluded from the LPS challenge and L^13^CU test due to health issues (i.e., diarrhea, rectal temperature exceeding 40 °C, lethargy, and antibiotic treatment). Consequently, analyses were conducted with samples from 38 experimental pigs, in the following four experimental treatment groups: HF-LPS, *n* = 9; HF-NaCl, *n* = 9; LF-LPS, *n* = 10; and LF-NaCl, *n* = 10 (Figure 1).

### Collection and Processing of Pig Saliva, Breath, Urine, and Fecal Samples

Saliva and breath samples were taken 30 and 15 min before (basal) and 1, 1.5, 2, 2.5, 3, 3.5, and 4 h and then hourly until 12 h and again 24 h after administration of the L^13^CU-labeled feed. Breath sampling was performed using a breath mask connected by a two-way valve to an aluminum-coated breath bag (0.3 L, FAN GmbH, Leipzig, Germany).^28^ Breath samples were transferred from the collection bags into 10 mL airtight screw cap glass tubes (Exetainer, Labco Ltd., Buckinghamshire, UK), preflushed with argon. The pigs’ mouths were rinsed 10 min before saliva collection to remove feed and debris, and the drinking water was turned off and feed dispensers closed. This procedure did not prevent the pigs from eating their full daily ration of feed. The saliva was collected by having the pigs chew on a salivary swab for 1–2 min (Salivette Cortisol, Sarstedt AG & Co. KG, Nümbrecht, Germany), held by a surgical clamp. Once the swab was moistened, it was placed in the Salivette tube (Sarstedt, Germany), stored on ice, and then centrifuged within 30 min of collection (4000*g*, 4 °C for 15 min). Saliva samples were stored at −20 °C until analysis. Urine collection was performed by placing metal trays beneath each metabolic crate, covered with a metal mesh to prevent contamination with feces, so that the urine could flow directly into ice-cooled 5 L buckets covered with another mesh. A basal urine sample (50 mL) was obtained from the overnight collection (12 h), which started 12 h prior to the L^13^CU administration and ended shortly before the L^13^CU administration (time point 0). Urine was pooled over 3 h intervals (0–3, 3–6, 6–9, and 9–12 h) and from 12 to 24 h post-L^13^CU to determine ^13^C and N excretion. The urinary weight was quantified (LF-LPS, *n* = 4; LF-NaCl, HF-NaCl, and HF-LPS, *n* = 3) to calculate the 24 h urine production (UP). After centrifugation (1000*g*, 10 min, 4 °C), the supernatant was transferred from the collection tubes to 15 mL tubes and stored frozen at −20 °C until analysis. Fresh feces were obtained by grab sampling (LF-LPS, *n* = 4; LF-NaCl, HF-NaCl, HF-LPS, *n* = 3) at each urine sampling time point. Samples were stored in 3 mL cryotubes at −20 °C until analysis to determine ^13^C and N excretion. The total fecal production (FP) was calculated based on the feces collected over 24 h. Baseline breath and saliva samples were obtained from three experimental pigs of the HF-NaCl and LF-NaCl groups (total *n* = 6) prior to the LPS challenge and the L^13^CU dose. The purpose was to normalize the ^13^CO_2_ enrichment in breath and saliva with respect to the natural ^13^C content of the experimental diets.

### Zootechnical Parameters

Daily feed intake (g/d) was determined by measuring the total feed offered in the morning of the experimental day and subtracting what remained the following morning in the feed dispenser. The DM (g/d) intake was derived from the daily feed intake multiplied by the DM of the respective diet. Total daily metabolizable energy intake (MJ/d) was calculated by multiplying the daily feed intake by the energy content of the diets. Daily water intake (L/d) was determined from meter readings taken at the start of (0 h) and 24 h after the L^13^CU test and corrected by the loss of water collected in a bucket connected directly to the water dispenser. Fecal consistency was recorded at d49, 50, 56, 57, 63, and 64 using a 5-grade rating protocol.^29^

### Analysis of Feed, Fecal, Urine, Breath, and Saliva ^13^C Abundance

The ^13^C abundances of the two experimental diets (LF, HF) and the ^13^C abundance in fecal (1.5 mg ground and predried aliquots), urinary (50 μL aliquots), and breath CO_2_ samples were analyzed in duplicate to calculate the ^13^C balance (^13^C ingested–^13^C excreted).^30^ Saliva (0.1 mL) was mixed with 0.1 mL of 10% lactic acid in 4 mL screw cap glass tubes, preflushed with argon, and incubated for at least 2 h at room temperature to release the CO_2_ from the matrix. Saliva CO_2_ samples were then analyzed using a continuous flow Gas Bench II coupled with isotope-ratio mass spectrometry (DELTA Plus XL; ThermoFinnigan, Bremen, Germany).

The ^13^C abundances in breath and saliva CO_2_ and urine and feces samples were expressed as delta values δ^13^C (‰) (relative to the reference δ^13^C value of the Pee Dee Belemnite Limestone standard). Delta values Δδ^13^C (‰) (enrichments) were calculated as the change in the δ^13^C of the test sample relative to the δ^13^C of the basal sample collected prior to L^13^CU administration. The δ^13^C values were converted to atom percentage values, reflecting the percentage of ^13^C atoms present in each sample, using published equations.^31^

### Calculation of Kinetic Parameters and OCTT from Breath and Saliva Samples

The Δδ^13^C enrichment–time curves for breath and saliva for each animal were subjected to curve-fitting software (TableCurve 2D v5.01, Cranes Software International Ltd.). The curve with the highest coefficient of determination that passed through zero was selected. The area under the enrichment-time-curve (AUC), maximum enrichment (*E*_max_), and time to reach the maximum enrichment (*T*_max_) were derived. The OCTT was derived from the time at which the ^13^C enrichment of CO_2_ in breath and saliva was significantly higher than the pretracer baseline value (+2 Δδ^13^C, ‰), as the mean SD × 2 was <2‰, in line with literature.^9^ The natural ^13^C abundance of the CO_2_ baseline (without tracer) was −24.2 ± 0.33 δ^13^C ‰ for breath and −15.9 ± 0.70‰ for saliva (mean ± SD).

### Urine, Fecal, and Feed DM, and Nitrogen, Carbon, and Urinary Urea Concentration

The DM of urine was determined in duplicate (tin capsules 5 mm × 9 mm, IVA-Analysentechnik GmbH and Co. KG, Meerbusch, Germany) at 55 °C for 16 h. Frozen fecal samples (3 g) and 20 g of each experimental feed were dried for 24 h in glass cups. After the samples were cooled to room temperature, all the samples were weighed and the DM content was calculated. The N and C content (% DM) in dried and finely ground feed and feces (tin capsules 3.3 × 5 mm), as well as in urine, were measured in duplicate using an elemental analyzer (Flash 1112 Series; Thermo Quest, Milan, Italy). Urea concentration in urine samples was determined by HPLC.^32^ The N ingested with the diet (IN), N excreted in feces (FN) and urine (UN), the total excreted nitrogen (Nex; i.e., FN + UN), and the UN/FN ratio over the 24 h test period were derived from the DM and N content (HF-LPS, HF-NaCl, LF-NaCl *n* = 3; LF-LPS *n* = 4). The FN and UN were also reported as a percentage of IN.

### Calculation of ^13^C Balance

The total ^13^C balance (as g/d = ^13^C input – ^13^C output) for the experimental groups (*n*, HF-LPS, HF-NaCl, LF-NaCl = 3; LF-LPS = 4) was determined as follows.

^13^C input calculations
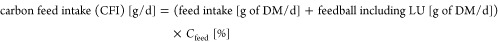


Then, the CFI expressed in moles, the ^13^C-AP of the feed, and ^13^C ingested with L^13^CU powder were used to derive the total ^13^C input
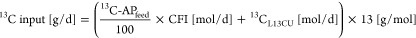


^13^C output calculations

We measured the ^13^C expelled via breath, urine, and feces over the 24-h test period. The rate of C exhaled in breath was calculated using unpublished data from our group on similarly aged pigs (d76), using a mean hourly CO_2_ production adjusted for kg BW [mean 24 h = 31.7 mmol/(kg·h)].
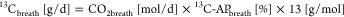


For feces (f) and urine (u), the following equations were used
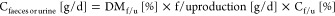

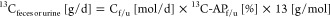

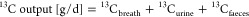

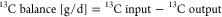


The ^13^C balance of each pig was compared among the treatment groups.

### Detection of *C. innocuum* in Feces

In humans, bacterial enzymes capable of degrading the sugar-urea bond of LU have been reported to originate from *C. innocuum* in the LI.^11^ We therefore were interested to know whether *C. innocuum* was also present in our experimental pigs. For this purpose, DNA was extracted from 91 frozen fecal samples (46 from pigs aged d75, at 0, 4, and 12 h relative to the LPS injection and 45 from pigs aged d76, at 0, 6, and 12 h relative to the L^13^CU administration) using the QIAamp Fast DNA Stool Mini Kit (Qiagen, Lot-No.: 175037509, Hilden, Germany) following manufacturer’s instructions. In total, samples of 4 LF-LPS and HF-LPS and 5 LF-NaCl and HF-NaCl animals were used. The DNA quantity was checked using a NanoDrop spectrophotometer (Thermo Fisher Scientific, Dreieich, Germany). Each reaction mix (10 μL) contained 2 μL of DNA template (20 ng/μL), 6.35 μL deionized water, 1 μL of Supratherm Buffer (10×), 0.05 μL of SupraTherm Taq DNA polymerase, and 0.2 μL of each primer and dNTP mixture. Specific primers of *C. innocuum* with an expected product size of 100 bp^33^ (forward: 5′-TTTGAAGCAGACCTCTTCCG-3′ and reverse: 5′-ATACAGCGGTATGCAGATTCC-3′) were used. In parallel, primers to amplify the 16S rRNA genes with an expected product size of 377 bp were used to verify the presence of microbial DNA (16SV4a-forward: 5′-AATGATACGGCGACCACCGAGATCTACACTAGCGAGTTATGGTAATTGTGTGBCAGCMGCCGCGGTAA-3′ and 16SV34a-reverse: 5′-CAAGCAGAAGACGGCATACGAGATAGTAGCGTAGTCAGTCAGCCGGACTACHVGGGTWTCTAAT-3′). Water was added to the reaction mix as a negative control. A positive control isolated from the feces of 8-week-old pigs was obtained from the German Collection of Cell Cultures (DMSZ, Braunschweig, Germany; *C. innocuum*, Smith and King 1962, DSM 1286). Thermocycling for *C. innocuum* specific primers reactions was carried out under the following conditions: 95 °C for 5 min, 40 cycles of 95 °C for 15 s, 57 °C for 30 s, and 72 °C for 30 s, with a final extension of 72 °C for 5 min. Additionally, thermocycling for 16S primers reactions was: 95 °C for 2 min, 35 cycles of 95 °C for 30 s, 50 °C for 60 s, and 72 °C for 90 s, with a final extension of 72 °C for 10 min. The amplification of the PCR products was checked in 3.5% agarose gels (+Ethidium bromide: 3.5 μL for 100 mL gel), and 10 μL of the PCR products with 1.5 μL of stop mix (6×) were run in a gel electrophoresis system, using a 50 bp DNA-marker (Carl Roth GmbH, Karlsruhe, Germany).

### Statistical Analysis

Model 1 was used to evaluate the test parameters urinary urea, UN, UP, NEX, FN, FP, IN, and ratio of UN/FN from feces and urine samples, and the ^13^C input–output balance was an ANOVA using the MIXED procedure of SAS (Version 9.4; SAS Institute Incorp., Cary, North Carolina, USA) that contained the fixed effect of diet (HF, LF), Trt (LPS, NaCl) and the interaction diet × Trt, and Sow as a random effect. The SLICE statement was used for performing partitioned analyses of the least-squares means for the diet × Trt interaction. Model 2 used for the evaluation of BW and fecal score was the same as Model 1 and contained the fixed effects of diet, Trt, Age, and the interaction diet × Trt × age. The SLICE statement was used for performing partitioned analyses of the least-squares means for the diet × Trt × age. Repeated measurements on the same animal at different ages were considered by the repeated statement of the MIXED procedure using the SUBJECT = animal option to define the blocks of the block diagonal residual covariance matrix and the TYPE = CS option to define their covariance structure. Model 3, used to evaluate the L^13^CU test parameters zootechnical data (EI, WI, FI) and breath and saliva AUC, *E*_max_, *T*_max_, and OCTT, was the Generalized Linear Mixed Model (GLIMMIX) procedure of SAS, using a Gaussian model (model statement: distribution = Gaussian, link = Identity), with the fixed effects of diet, Trt, and the interaction diet × Trt, and sow as a random effect. The SLICE statement was used for performing partitioned analyses of the least-squares means for the diet × Trt interaction. Model 4, used for the evaluation of breath and saliva Δδ^13^CO_2_, was the same as Model 3 but included the fixed effect of time with repeated measurements on the same animal taken into account using the SUBJECT = animal option to define the blocks of the block-diagonal residual covariance matrix and the TYPE = SP (EXP) option to define their covariance structure. The SLICE statement was used for performing partitioned analyses of the least-squares means for the diet × Trt × time interaction. Model 5, used for comparing breath and saliva AUC, *E*_max_, *T*_max_, and OCTT, was the same as Model 3 but included the fixed effect of Matrix (breath, saliva). The SLICE statement was used for performing partitioned analyses of the least-squares means for the diet × Trt × matrix interaction.

Model selection was based on Akaike’s information criterion.^34^ Sow was defined as a random factor, which allowed modeling of littermates from the same sow and inference about the fixed effects, and piglet was the experimental unit. Normality was tested using the Shapiro–Wilks test and Q–Q Plots (SAS), and when data met the assumptions of normal distribution, differences were assessed using the Tukey–Kramer test. All data in this study met the conditions of normality. Pearson correlations were computed between the breath and saliva Δδ^13^CO_2_, OCTT, *E*_max_, *T*_max_ and AUC. A Bland–Altman analysis was used to assess the comparability between breath and saliva measurements of OCTT and the limits of agreement (LoA).^35^ Differences for all tests were considered significant at *p* < 0.05.

## Results

### Zootechnical Parameters

At d 76 of age (L^13^CU test), the diet affected feed and DM intake (*p* < 0.001). Pigs fed the HF diet had a higher feed (1294 ± 27.8 vs 1238 ± 22.9 g/d; *p* < 0.001) and a higher DM intake (1169 ± 20.2 vs 1101 ± 20.2 g/d; *p* < 0.001) but a similar energy intake compared to the LF pigs. The feed and DM intake were higher for the HF-LPS (1280 ± 31.6; 1167 ± 20.2 g/d) and HF-NaCl groups (1308 ± 24.0; 1170 ± 20.2 g/d) than for the LF-LPS (1233 ± 25.2; 1100 ± 20.2 g/d, *p* < 0.001) and LF-NaCl (1243 ± 20.7; 1101 ± 20.2 g/d) groups, respectively. Diet did not affect the fecal score of HF and LF fed pigs prior to the L^13^CU test (data not shown, *p* > 0.1).

### ^13^C Enrichment in Breath, Saliva, Urine, and Feces

**Figure 2:**
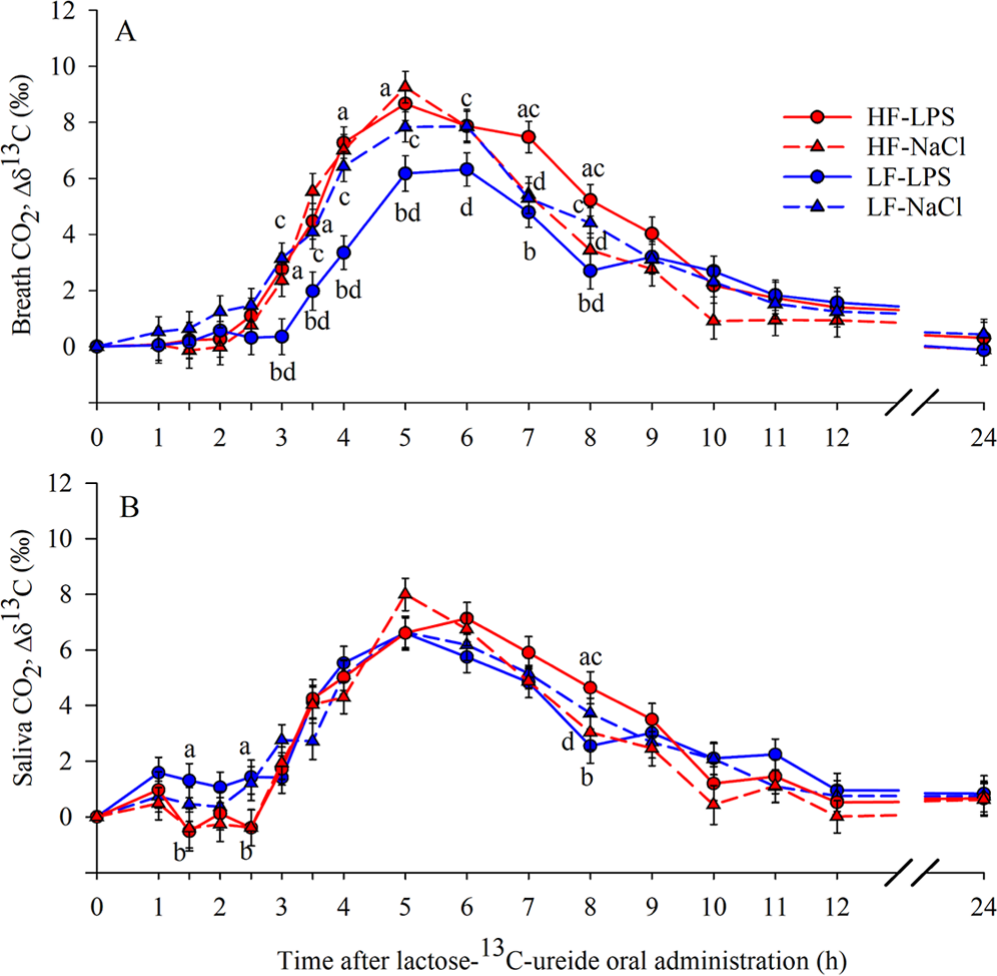
Breath and saliva ^13^C enrichment in CO_2_ after ingestion of 400 mg L^13^CU in 76 d-old pigs. (A) Breath Δδ^13^CO_2_ (‰), and (B) saliva Δδ^13^CO_2_ (‰) enrichments over time, from 0 to 24 h in HF-LPS (*n* = 7–9), HF-NaCl (*n* = 8–10), LF-LPS (*n* = 8–10) and LF-NaCl (*n* = 8–9) experimental groups. Values are least-squares means ± standard error of the mean. (a, b) indicate significant differences within time point between diets within treatment. (c, d) indicate significant differences within time point between treatments within diet (*p* < 0.05).

The factors diet (*p* < 0.01), time, and the interactions diet × Trt, diet × time (*p* < 0.001) and Trt × time (*p* < 0.05) affected the Δδ^13^C values of breath CO_2_ (Figure 2A). Pigs fed the HF diet had higher Δδ^13^C values than the LF pigs (3.19 vs 2.73 ± 0.18‰; *p* < 0.01). From 3 to 5 h and at 7 and 8 h after L^13^CU administration, the breath ^13^CO_2_ enrichment of LF-LPS pigs was lower than that of HF-LPS group (*p* < 0.05; Figure 2A). From 3 to 6 h and at 8 h, the breath CO_2_ of the LF-LPS group was less enriched with ^13^C compared to the LF-NaCl group (*p* < 0.05; Figure 2A). The level of Δδ^13^C found in breath CO_2_ of the HF-LPS group was higher than that of the HF-NaCl group at 7 and 8 h after L^13^CU administration (*p* < 0.05; Figure 2A).

We observed an effect of time (*p* < 0.001) and diet × time interaction (*p* < 0.05) on salivary Δδ^13^C values (Figure 2B). The HF-LPS group had lower ^13^C enrichment at 1.5 and 2.5 h (*p* < 0.05) and higher ^13^C enrichment after 8 h (*p* < 0.01), compared with the LF-LPS group (Figure 2B). At 8 h after L^13^CU administration, the salivary CO_2_ of the HF-LPS group was more ^13^C enriched compared to the HF-NaCl group (*p* < 0.05; Figure 2B).

**Figure 3:**
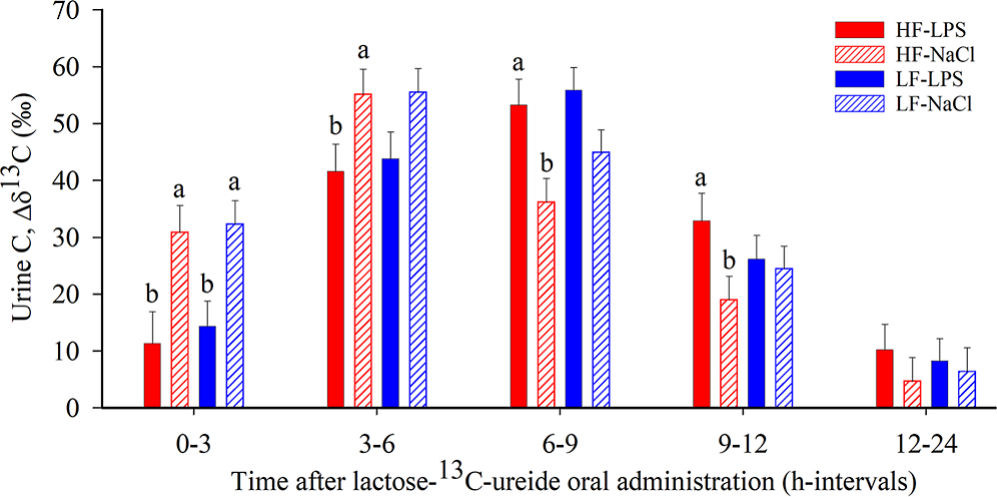
Urine ^13^C enrichment after ingestion of 400 mg L^13^CU in 76 d-old pigs. Different bars for each experimental group (HF-LPS, *n* = 6–9; HF-NaCl, *n* = 7–9; LF-LPS, *n* = 7–10; and LF-NaCl, *n* = 9–10) represent the average ^13^C enrichment of urine collected during consecutive time intervals. Values are least-squares means ± standard error of the mean. (a, b) indicate significant differences within time interval between treatments within diet (*p* < 0.05).

The ^13^C enrichment of urine was affected by time and the interaction of time × Trt (*p* < 0.001) (Figure 3). The urinary Δδ^13^C enrichment of pigs in the HF-LPS and LF-LPS groups compared to those of the HF-NaCl and LF-NaCl groups were lower from 0 to 3 h (*p* < 0.01) and from 3 to 6 h (*p* < 0.05; Figure 3). Urinary ^13^C enrichment was higher in the HF-LPS group than in the HF-NaCl group after 6 to 9 h (*p* < 0.01) and 9 to 12 h (*p* < 0.05; Figure 3).

The ^13^C enrichment in the fecal samples was in the range of the natural ^13^C abundance (Δδ^13^C ‰ min–max range: HF-LPS, 0–0.31; HF-NaCl, 0–0.24; LF-LPS, 0.05–0.52; and LF-NaCl, 0–0.45).

### OCTT and Kinetic Parameters Calculated from ^13^CO_2_ Enrichments in Breath and Saliva Samples

The OCTT (*p* < 0.05) and ^13^CO_2_*E*_max_ (*p* < 0.01) measured in breath were affected by the interaction diet × Trt, and the ^13^CO_2_*E*_max_ (*p* < 0.01) was influenced by Trt (Table 3). The LPS-treatment resulted in a lower ^13^CO_2_*E*_max_ (8.47 ± 0.48 vs 10.90 ± 22.42; *p* < 0.01) than the NaCl-treatment. The OCTT of the HF-LPS group was shorter compared to LF-LPS (*p* < 0.05). Within the LF group, LPS-treated pigs had a longer OCTT than the NaCl controls (*p* < 0.05). Breath ^13^CO_2_*E*_max_ was lower in LF-LPS than HF-LPS (*p* < 0.05) and LF-NaCl groups (*p* < 0.001). In saliva, the only difference observed was for AUC, which was larger for the LF-LPS group compared to the LF-NaCl group (*p* < 0.05, Table 3).

**Table 3:** Breath and Saliva OCTT and Kinetic Parameters of Pigs at 76 Days of Age Fed Low Fibre or High Fibre Diets and Pre-challenged with LPS or NaCl, during the Lactose-^13^C-Ureide Test^a^

items^b^^,^^c^	low fiber		high fiber		*p* values^d^		
	LPS	NaCl	LPS	NaCl	Diet	Trt	Diet × Trt
Breath							
OCTT, h	3.42 ± 0.21ac	2.88 ± 0.21d	2.86 ± 0.23b	3.21 ± 0.22	0.505	0.591	<0.05
*E*_max_, Δδ^13^C ‰	7.27 ± 0.70bd	11.9 ± 0.75c	9.68 ± 0.65a	9.86 ± 0.61	0.807	<0.01	<0.01
*T*_max,_ h	5.63 ± 0.31	5.20 ± 0.30	5.00 ± 0.33	5.16 ± 0.32	0.240	0.647	0.291
AUC, Δδ^13^C ‰·h	45.7 ± 2.90	46.7 ± 2.60	48.6 ± 2.80	46.4 ± 2.60	0.545	0.802	0.517
Saliva							
OCTT, h	3.04 ± 0.22	3.34 ± 0.22	2.87 ± 0.27	3.34 ± 0.23	0.718	0.111	0.699
*E*_max,_ Δδ^13^C ‰	7.12 ± 0.87	7.85 ± 0.75	7.86 ± 0.80	8.69 ± 0.75	0.297	0.304	0.944
*T*_max_, h	5.95 ± 0.34	5.17 ± 0.41	5.19 ± 0.36	5.14 ± 0.34	0.281	0.251	0.314
AUC, Δδ^13^C ‰·h	50.9 ± 4.30c	34.9 ± 4.60d	40.0 ± 4.80	40.1 ± 5.20	0.533	0.106	0.102

a Abbreviations: AUC, area under the enrichment–time curve; *E*_max_, maximum enrichment; LPS, lipopolysaccharide challenge; NaCl, sodium chloride (control group); OCTT, oro-cecal transit time; *T*_max_, time to reach the maximum enrichment; Trt, treatment (LPS or NaCl control).

b Values are expressed as least-squares means and standard error of the mean: LF-LPS/HF-NaCl *n* = 6–9; LF-NaCl *n* = 6–10; HF-LPS *n* = 6–8.

c Values with a or b lower case letters within row differ between diets within treatment (*p* < 0.05). Values with c or d lower case letters within row differ between treatments within diet (*p* < 0.05).

d *F*-test.

The ^13^CO_2_ AUC and *E*_max_ were affected by the sample matrix (*p* < 0.05) and diet × Trt × matrix interaction (*p* < 0.05). The breath ^13^CO_2_ AUC and *E*_max_ were greater (47.0 vs 41.9 ± 2.4 Δδ^13^C ‰·h, *p* < 0.05; 9.67 ± 0.5 vs 7.87 ± 0.4 Δδ^13^C ‰, *p* < 0.001) than in saliva. The OCTT from breath and OCTT from saliva did not differ (*F*-test *p* > 0.17). However, in the LF-NaCl group, the OCTT measured in saliva was longer (3.28 vs 2.86 ± 0.21 h) than in the breath samples (*p* < 0.05), while ^13^CO_2_ saliva AUC (36.4 ± 3.68 vs 47.5 ± 3.51 Δδ^13^C ‰·h) and *E*_max_ (7.94 ± 0.69 vs 11.9 ± 0.83 Δδ^13^C ‰) were smaller than those from breath ^13^CO_2_ (*p* < 0.05).

### Pearson Correlation and Bland–Altman Analysis between Breath and Saliva Measurements

Saliva Δδ^13^C values of all four experimental groups correlated positively with the breath values (*p* < 0.001), with all correlation coefficients being above 0.90 (Table S3). The Pearson correlation between breath and saliva OCTT for HF-NaCl, LF-NaCl and HF-LPS (*r* > 0.70; *p* < 0.05) was positive but not for the LF-LPS group. Breath and saliva *E*_max_ correlation was positive for HF-LPS, LF-LPS, and LF-NaCl (*r* > 0.80; *p* < 0.01), while breath and saliva *T*_max_ were positively correlated in all four groups (*r* > 0.87; *p* < 0.01). The correlation between breath and saliva AUC was positive for HF-LPS and LF-NaCl (*r* > 0.80; *p* < 0.01; Table S3).

**Figure 4:**
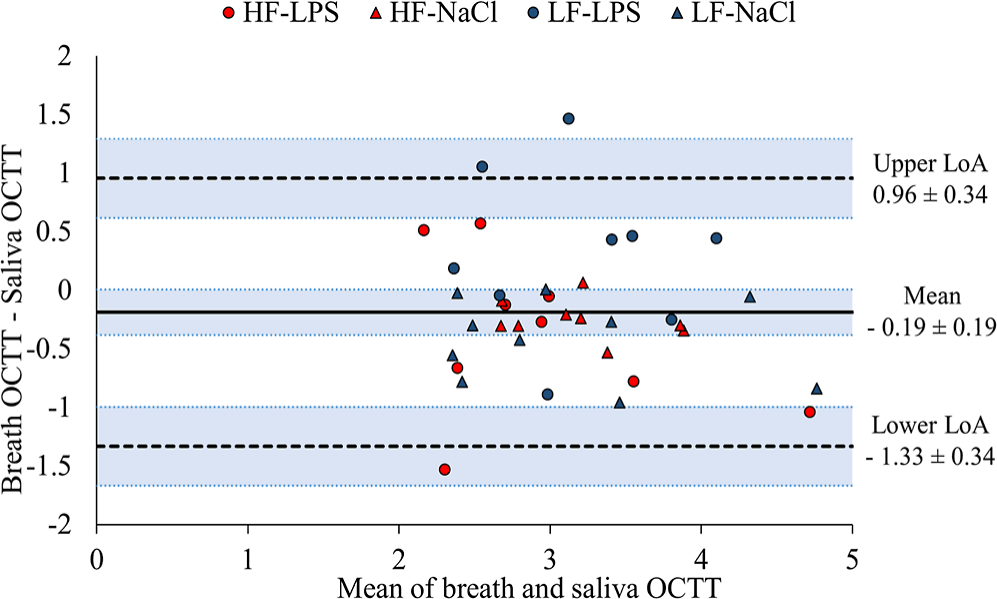
Bland–Altman plot comparing breath and saliva measurements for OCTT. Plot of differences between breath OCTT and saliva OCTT vs the mean of the two measurements for all the experimental groups (HF-LPS, HF-NaCl and LF-LPS, *n* = 9; LF-NaCl, *n* = 10). The solid black line represents the mean difference (−0.19) ± 0.19 of 95% confidence interval (CI) represented by the light blue shaded region delimited by blue dotted lines. The black dashed lines indicate the upper (0.96) and lower (−1.33) LoA ± 0.34 CI.

The agreement of OCTT measured in breath and saliva samples, assessed using a Bland–Altman plot (Figure 4), showed a mean difference of −0.19 h, with a 95% confidence interval ranging from −0.38 to 0.007 h, indicating that saliva OCTT was always somewhat longer than breath OCTT. The LoA was between −1.33 (lower LoA) and 0.96 (upper LoA) ± 0.34 (95% confidence interval). The majority (91.9%) of 37 comparisons between breath and saliva OCTT were within the upper and lower LoA, while 8.11% were outside the LoA (Figure 4).

### Urine and Feces Production, N Balance, and ^13^C Balance

At d 76, the FP, UP, IN, FN, and UN/IN (*p* < 0.05) were affected by diet, while FP, UP, UN, and UN/IN (*p* < 0.05) were influenced by Trt (Table 4). The groups fed the HF diet had greater FP (931 ± 50.5 vs 503 ± 47.2 g/d, *p* < 0.001) but lower UP (1439 ± 364 vs 2956 ± 341 g/d, *p* < 0.05) than those fed the LF diet.

**Table 4:** Feces and UP, ^13^C and Nitrogen Excretion of Pigs at 76 Days of Age Fed Low with Fibre or High Fibre Diets and Challenged with LPS or NaCl, during the Lactose-^13^C-Ureide Test (24 h)^a^

items^b^^,^^c^^,^^d^	low fiber		high fiber		*p* values^e^	
	LPS	NaCl	LPS	NaCl	diet	Trt
FP, g/d fresh	429 ± 61.9b	577 ± 71.4b	774 ± 71.4ad	1089 ± 71.4ac	<0.001	<0.01
UP, g/d fresh	1997 ± 446d	3915 ± 515ac	895 ± 515	1984 ± 515b	<0.05	<0.05
^13^C balance, g/d	1.68 ± 0.33	1.57 ± 0.38	1.60 ± 0.38	1.81 ± 0.38	0.829	0.889
IN, g/d	31.4 ± 0.57b	32.2 ± 0.66b	38.6 ± 0.66a	39.5 ± 0.66a	<0.001	0.196
FN, g/d	5.07 ± 0.77	6.20 ± 0.89b	6.97 ± 0.89	9.24 ± 0.89a	<0.05	0.081
FN/IN, %	16.0 ± 2.28	19.3 ± 2.63	18.1 ± 2.63	23.4 ± 2.63	0.253	0.128
UN, g/d	8.93 ± 0.76c	6.26 ± 0.88d	7.11 ± 0.88	5.91 ± 0.88	0.234	<0.05
UN/IN, %	28.6 ± 2.36ac	19.4 ± 2.72d	18.4 ± 2.72b	15.0 ± 2.72	<0.05	<0.05
Nex, g/d	14.0 ± 0.65	12.5 ± 0.75a	14.1 ± 0.75	15.1 ± 0.75b	0.087	0.744
UN/FN	2.09 ± 0.34	1.12 ± 0.39	1.02 ± 0.39	0.65 ± 0.39	0.072	0.108

a Abbreviations: FN, fecal nitrogen; FN/IN, fecal nitrogen as % IN; FP, fecal production; IN, ingested nitrogen; LPS, lipopolysaccharide challenge; Next, nitrogen excretion (FN + UN); NaCl, sodium chloride (control group); Trt, treatment (LPS challenge or NaCl control); UN, urine nitrogen; UN/IN, urine nitrogen as % IN; UP, urine production.

b Values are least-squares means ± standard error of the mean: LF-LPS *n* = 4; LF-NaCl, HF-LPS, HF-NaCl *n* = 3.

c Unless otherwise stated, values were calculated on DM basis.

d Values with a or b lower case letters within row differ between diets within treatment (*p* < 0.05). Values with c or d lower case letters within row differ between treatments within diet (*p* < 0.05).

e *F*-test, diet × Trt interaction was not significant (*p* > 0.1).

The groups fed the HF diet had greater IN (39.0 ± 0.46 vs 31.8 ± 0.43 g/d, *p* < 0.001) and FN (8.10 ± 0.63 vs 5.64 ± 0.59 g/d, *p* < 0.05) but lower UN/IN (16.7 ± 1.93 vs 24.0 ± 1.80%, *p* < 0.001) than the LF-fed. The LPS-treated groups showed reduced FP (601 ± 47.2 vs 833 ± 50.5 g/d, *p* < 0.01) and UP (1446 ± 341 vs 2950 ± 364 g/d, *p* < 0.05) but greater UN (8.02 ± 0.58 vs 6.08 ± 0.58 g/d, *p* < 0.05) and UN/IN (23.5 ± 1.80 vs 17.2 ± 1.93%, *p* < 0.05) compared to the NaCl-treated groups. The FP of the HF-LPS group was greater than that of the LF-LPS group (*p* < 0.01) but lower than that of the HF–-NaCl controls (*p* < 0.05; Table 4). The FP was greater for the HF-NaCl group than for LF-NaCl (*p* < 0.001). The LF-LPS and HF-NaCl groups had lower UP compared to the LF-NaCl group (*p* < 0.05). The HF-LPS and HF-NaCl groups had a higher IN than the LF-LPS and LF-NaCl groups (*p* < 0.001).

The FN was higher for the HF-NaCl group compared to the LF-NaCl group (*p* < 0.05). The UN and UN/IN of the LF-LPS group were higher than that of the LF-NaCl controls (*p* < 0.05), and the HF-LPS group showed a smaller UN/IN ratio than the LF-LPS group (*p* < 0.05). The Nex of HF-NaCl group was higher than that of LF-NaCl group (*p* < 0.05; Table 4). The factors time, Trt, and the interaction diet × time × Trt (*p* < 0.001) affected the urea concentrations in urine samples (Table S4).

Overall, urinary urea concentration was higher in LPS-treated groups (200 vs 95.7 ± 15.4 mmol/L, *p* < 0.001) than in the NaCl-groups. Urine collected overnight (−12 to 0 h), prior to L^13^CU administration from LF-LPS (*p* < 0.001) and HF-LPS (*p* = 0.002), had a higher urea concentration than that from LF-NaCl and HF-NaCl, respectively. In the interval from 0 to 3 h, the urea concentration was higher in LF-LPS than in LF-NaCl (*p* < 0.001) and HF-LPS (*p* = 0.03). Between 3 and 6 h, urea concentration was higher in HF-LPS than in HF-NaCl (*p* < 0.001) and LF-LPS (*p* = 0.003). For the interval 6–9 h, the urine of LF-LPS (*p* = 0.02) and HF-LPS (*p* = 0.004) had a higher urea concentration than that of LF-NaCl and HF-NaCl, respectively (Table S4).

### Fecal Microbiota

The PCR results from fecal samples collected the day before (d75; Figure S2) and on the day of the L^13^CU test (d76; Figure S3) show that all samples contained bacterial DNA (Figures S2 and S3, panel B), but *C. innocuum* could only be detected in fecal samples from one LF-LPS and two LF-NaCl pigs (Figure S3, Panel A).

## Discussion

In pigs, small intestinal transit studies are highly invasive if they involve the cannulation of different gut segments.^3^ This is the first study to use L^13^CU to estimate OCTT in the breath of pigs, and the results indicate that the method worked, as evidenced by a significant increase of ^13^CO_2_ enrichment exceeding 2 Δδ^13^C ‰-units above the natural ^13^C baseline. The average OCTT obtained (3.09 ± 0.23 h) was similar to OCTT observed in humans^9^ and horses (3.24 ± 0.65 h).^13^ Using the same method, other human studies have reported a longer average OCTT (4.86 ± 0.97, and 5.8–6.0 ± 2 h),^10,36^ due to differences between subjects and study design. The second aim of this study was to investigate the effects of different levels of DF and an LPS challenge on GI parameters observed with the L^13^CU breath test. The OCTT and *E*_max_ values were comparable between the control (NaCl) groups fed HF or LF diets. However, the LF-LPS group exhibited a longer OCTT and a lower ^13^CO_2_*E*_max_ compared to the HF-LPS and LF-NaCl groups. In addition, the LPS challenge affected the course of the ^13^C excretion in the urine, and the level of DF influenced the N metabolism. The OCTT measured in saliva ^13^CO_2_ vs breath ^13^CO_2_ was similar, suggesting the principal applicability of the saliva method. The *C. innocuum*, reported to be responsible for the cleavage of the sugar-ureide bond in humans, was only detected in a few fecal samples, indicating that other bacterial species might be responsible in pigs.

### Effects of DF Level on OCTT Measured in Breath CO_2_

Soluble fibers (i.e., sugar beet pulp, pectin, or guar gum) are associated with slower gastric emptying rate, increased viscosity, satiety, and transit time.^17,37^ In contrast, insoluble fibers (i.e., lignin and cellulose) and starch rather increase the digesta flow rate thereby reducing transit times.^17^ Our HF diet was based on rye, wheat, and corn, which was enriched with sugar beet pulp and lignocellulose, a mixture of soluble and insoluble fibers. As in previous studies,^38^ the lower energetic content of the HF diet compared to the LF diet (rye-wheat-corn diet enriched with wheat starch) was compensated for by increasing the feed allowance and DM intake in the HF-group compared to the LF-group. No difference in OCTT was observed between control (NaCl) HF and LF pigs, suggesting the additional fiber had no effect on L^13^CU transit time. Therefore, the effects of the two different fiber sources in the HF diet on the upper GI transit canceled each other out, which seems to be explained by the similar ratio between soluble and insoluble NSP in the HF (29.2%:70%) and LF (22.9%:77%) diets. Another reason for the lack of difference in OCTT could be that DF mostly affects the passage rate in the LI of pigs^39^ and to a lesser extent in the SI.^40^ Previous research on cannulated pigs^41^ demonstrated that increasing dietary NSP through the addition of palm kernel expeller and toasted soybean hulls had no effect on SI transit time which was determined as the difference between the transit time of 10% Cr_2_O_3_ and BaSO_4_ between proximal jejunum and terminal ileum.^41^ Although directly comparing our OCTT results with previous research is challenging due to the different definitions for intestinal transit parameters, we argue that comparing them with a marker’s time of first appearance is more appropriate than with the mean retention time (MRT, average time digesta spends within a gut segment). This perspective is supported by another cannulation study in pigs,^16^ in which the authors reported that increasing the insoluble fiber content (wheat bran 0, 200, or 400 g/kg) had no effect on the time of first appearance of solid (2.5, 2.2, 2.3 h) or liquid (2.9, 2.3, 2.8 h) markers in the ileal digesta, while the MRT between the duodenal and ileal cannulas was reduced with 400 g/kg wheat bran inclusion. In contrast to our results, a cannulation study in 27 kg BW pigs reported that the addition of 7% guar gum (soluble) or 7% cellulose (insoluble) to a control diet at the expense of corn-starch prolonged the time to the first appearance of Cr_2_O_3_ in the distal ileum (control 2.15 vs fiber 3.17 h).^42^ Additionally, in ileal-cannulated pigs fed with an oat-based diet (insoluble fiber), the first appearance of a whole digesta marker (TiO_2_) was later than for a rice-based diet (oats 3.89 vs rice 2.66 h), while there were no differences for the fibrous phase of digesta marker (Cr-mordanted wheat bran; oats 3.71 vs rice 2.82 h).^37^ Previously, it has been shown^38^ that total tract MRT of solids was linearly related to DM intake, but in our study, the higher DM intake in HF pigs did not shorten OCTT using the L^13^CU method. Overall, the discrepancies in SI transit time parameters observed across different studies result from a combination of factors, including variations in the quantity, type, and source of DF, particle size, type and location of cannulas, measurement methods, and the various definitions for transit time parameters. The observation that pigs in the HF and LF NaCl-control groups did not differ in OCTT may also indicate that there was little variability in the presence and activity of the LI bacteria responsible for the LU bond cleavage, as previously hypothesized.^43^

### Effects of LPS on OCTT and ^13^CO_2_ Kinetics in Breath CO_2_

Administration of LPS 24 h prior to the L^13^CU test resulted in a delayed increase of the ^13^CO_2_ enrichment and lower *E*_max_ in the breath of LF pigs, which was associated with a longer OCTT compared to LF-NaCl and HF-LPS pigs. In mice,^44^ dogs,^45^ and horses,^46^ LPS had inhibitory effects on GI motility.^19^ It was reported that reduced intestinal motility could be a consequence of toll-like receptors activation (present in smooth muscle cells of the GI tract) following an LPS challenge.^47^ In addition, a reduced ability of SI to transport and absorb nutrients in pigs^48^ and a reduced substrate oxidation and cellular energy status in piglet SI mucosae were reported 24 h after LPS exposure.^49^ In vitro studies on human colonic mucosa and colonic smooth muscle cells exposed to LPS showed reduced muscle cell contractility.^50,51^ Therefore, these observations suggest that the GIT of the LF pigs used in the present study had not fully recovered 24 h post-LPS administration when the L^13^CU test was initiated and that the HF diet may have had a “protective” effect against LPS induced functional disorders. Whether this was associated with changes in the intestinal microbiota composition remains to be investigated, but it was reported that LPS can alter the colonic^52^ or jejunal microbiota and inhibit carbohydrate and energy metabolism-related pathways.^53^

Our observation that LPS reduced OCTT in pigs fed the LF diets but not in pigs fed the HF diets may be due to a lower production of SCFAs in the LF pigs. In the SI, SCFAs can already be produced from soluble fiber, while the insoluble fibers are mostly fermented in the LI.^54^ The SCFAs play a crucial role in mitigating intestinal inflammation and maintaining mucosal integrity,^54^ regulating not only colonocyte proliferation and growth but also stimulating cell proliferation and growth of the SI.^55^ One study demonstrated how butyrate could maintain transepithelial electrical resistance in SI porcine cells (IPEC-J2) at 24-h postin vitro LPS-induced damage,^56^ highlighting its protective role in gut barrier integrity. Others^57^ reported that pig ileal concentration of SCFAs was higher when alfalfa fiber was used compared to a control diet, 4-h after an LPS challenge. Wellington et al.^58^ showed a reduced barrier function during the LPS period in LF-fed pigs but not in HF-fed pigs which was associated with increased goblet cell number and fecal mucin output. Therefore, we assume that the lack of LPS effect on OCTT in HF-fed pigs results from the presumably higher production of SCFAs which alleviates the inflammatory response associated with reduced intestinal smooth muscle contractility^50^ and/or changes in the microbial composition.^53^

### Effects of the DF Level and LPS on Lactose-Ureide Carbon Excretion and Nitrogen Metabolism

To shed some light on the reasons for the course and extent of ^13^C excretion in urine and feces, we determined the total FP and UP during the OCTT test. In line with the previous research, FP was higher and UP was lower, for HF-NaCl vs the LF-NaCl pigs.^59^ This resulted in a higher FN content, which possibly derived from the higher IN of the HF group, as a consequence of their higher feed intake to balance for the lower energetic content or from the higher microbial protein synthesis in the LI, as previously shown for diets with higher fiber content.^60^ The UN excretion (as % of IN) of the LPS-treated groups was lower in the HF than in the LF groups, probably indicating the shift in N excretion from urinary urea to bacterial protein in feces, typical for fibrous diet.^60^ Taken together, these results combined with the observation of similar breath ^13^CO_2_ enrichments and OCTT in the HF and LF NaCl-control groups indicate that fiber addition mainly act at the LI level rather than in the SI compartment.

The fate of L^13^CU after passing the SI was also monitored by measuring the level of ^13^C enrichment in urine and fecal samples. We observed that the ^13^C *E*_max_ in the urine of HF-NaCl and LF-NaCl groups was reached between 3 and 6 h and decreased after 6 h, which is consistent with previous findings in humans.^9^ In the LPS groups, the urinary ^13^C enrichment during the 0–3 h interval was lower and the urinary ^13^C *E*_max_ was reached later in the 6–9 h interval, compared to the NaCl-treated controls. If the ^13^C label is excreted in urine as the intact G^13^CU molecule, the present findings suggest that LPS delayed the release of G^13^CU in urine possibly caused by changes of the microbiome and/or liver or kidney function impairment.^61^

During the test period (24 h), the ^13^C enrichment detected in the feces was unaffected. Two reasons could be responsible for this finding. Since the observation period for ^13^C excretion was 24 h after administration of L^13^CU, it is likely that unmetabolized L^13^CU or G^13^CU has not yet been excreted, as the digesta MRT of pigs can range from 26 to 44 h.^16^ Alternatively, the ^13^C dose remaining in the LI or the ^13^C that could be bound in microbial protein is so low in relation to the mass of the unlabeled ^12^C that the fecal enrichment corresponds to the natural ^13^C abundance. Nevertheless, we have calculated the recovery of the ^13^C from the L^13^CU dose and the ^13^C in the consumed feed carbon over 24 h in breath, urine, and feces and determined that 55.5% of ^13^C left the body via breath and 2.3% via urine and 14.8% via feces. Our results also show a higher urinary urea concentration in the LPS-treated pigs, which confirms the results of another LPS pig model showing increased ureagenesis, indicating increased degradation of body protein, amino acid oxidation, and nitrogen loss.^62^ A limitation for the interpretation of the UP, FP, N and fecal ^13^C data is the small number of animals (*n* = 3–4) albeit results do agree with literature.

### Cleavage of the Sugar–Ureide Bond

In order for ^13^CO_2_ to be released from L^13^CU, the sugar–urea bond must be cleaved and the urea must be split. Microbial enzymes in the LI and not endogenous enzymes of the SI are responsible for the cleavage of the sugar-ureide bond.^12^ In the human cecum, this bond is cleaved by the bacterial species *C. innocuum*.^11^ In the present study, *C. innocuum* was detected in the fecal DNA of only three animals, suggesting another *Clostridium* or other bacteria species may be responsible for breaking the sugar–ureide bond in pigs. But since microbial composition in the feces do not reflect the total composition of the gut microbiome,^63^*C. innocuum* or other bacterial species able to split the sugar–ureide bond may occur in the cecum of the pigs. Despite the similarities shared by the gut microbiota of humans and pigs, their GI bacterial composition also presents some differences due to age, nutrition, and environmental factors.^64^ For this reason, it cannot be excluded that microbes in the terminal SI of pigs may contribute to a larger degree to the formation of G^13^CU from L^13^CU than in humans. It is however unlikely that ^13^C-urea derived from L^13^CU is degraded in the SI because microbial urease activity is negligible in the SI of pigs.^65^ More in-depth analyses of the intestinal microbiota in pigs given L^13^CU are needed to identify bacteria species responsible for breaking the sugar–ureide bond.

### Saliva as a Noninvasive Sample Matrix for Measuring OCTT

The use of saliva to measure OCTT with the L^13^CU method was also investigated due to its lower invasiveness compared to sampling breath.^15^ Saliva can accumulate various components circulating in the blood by passive diffusion, active transport, or filtration.^66,67^ Therefore, we hypothesized that a portion of the ^13^CO_2_ released into the bloodstream following the hydrolysis of the L^13^CU bond would also appear in saliva. Our results show that the Δδ^13^C and *T*_max_ data measured in saliva samples correlated positively with those of breath samples, although the OCTT, AUC, and *E*_max_ of saliva were not significantly correlated with the parameters derived from breath in at least one experimental group. The Bland–Altman plot showed more than 90% of the OCTT measurements to be in agreement between breath and saliva, supporting the use of saliva and breath interchangeably for assessing OCTT. Nevertheless, the saliva results showed only a few of the ^13^C kinetic differences between the experimental groups found in the breath. Explanations include the viscosity of saliva^66^ and possible addition of CO_2_ due to the increased bicarbonate production by the salivary glands due to chewing.^68^ The greater interindividual variability and baseline levels of ^13^C in saliva, compared to breath, along with the necessary sample preparation for CO_2_ extraction (including centrifugation, thawing, and acidification) may also have obscured potential differences. Therefore, future investigations into the potential of pig saliva as a noninvasive matrix should focus on optimizing the collection methods, refining sample processing, and validating salivary biomarkers.

The results of the present study demonstrate the potential of the L^13^CU breath test as a less-invasive method for monitoring aspects of GIT health and physiology in pigs. Our results also provide new insights into the effects of LPS stimulation of the immune system and its interactions with DF on components of gut functionality but still need to be independently validated in infection models. Additionally, saliva was identified as a suitable alternative to breath samples, aligning with the 3Rs principle (replace, reduce, and refine) but needs further validation.

## Supporting Information Available

Pretrial ^13^CO_2_ enrichment curves after oral lactose–^13^C-ureide (L^13^CU) administration measured in breath (A) and saliva (B) CO_2_; nutrient and energy contents of diets fed to sows and experimental piglets; pretrial experimental setup for assessing dosages and sampling times for the lactose–^13^C-ureide (L^13^CU) test in breath and saliva; correlation coefficients (*r*) and *p*-values between breath and saliva Δδ^13^C, OCTT, *E*_max_, *T*_max_, and AUC of the experimental groups; urea concentration of urine samples; agarose gel electrophoresis of PCR-amplified fragments of microbial DNA extracted from fecal samples collected on the LPS-challenge day (d75); and agarose gel electrophoresis of PCR-amplified fragments of microbial DNA extracted from fecal samples collected on the lactose–^13^C-ureide (L^13^CU) test day (d76) (PDF)

## List of Abbreviations

^13^C: heavy stable carbon isotope
AP: atom percent
AUC: area under the enrichment–time curve
BW: bodyweight
C: carbon
CO_2_: carbon dioxide
DM: dry matter
DNA: deoxyribonucleic acid
*E*_max_: maximum enrichment
FN: fecal nitrogen
FP: fecal production
G^13^CU: glucose–^13^C-ureide
GI: gastrointestinal
GIT: gastrointestinal tract
HF: high fiber
HPLC: high performance liquid chromatography
IN: ingested nitrogen
L^13^CU: lactose–^13^C-ureide
LF: low fiber
LI: large intestine
LPS: lipopolysaccharide
LU: lactose–ureide
MRT: mean retention time
N: nitrogen
NSP: nonstarch polysaccharides
NaCl: sodium chloride (saline solution control group)
Nex: total nitrogen excreted with urine and feces
OCTT: oro-cecal transit time
PCR: polymerase chain reaction
SCFAs: short chain fatty acids
SI: small intestine
*T*_max_: time to reach the maximum enrichment
Trt: treatment (LPS/NaCl)
UN: urinary nitrogen
UP: urinary production
Δδ^13^C: delta ^13^C values
δ^13^C: delta ^13^C values
