# Supplementary material for: Measurement of Oro-Cecal Transit Time in LPS-Treated Pigs Fed High and Low Fiber Diets Using the Lactose-13C-Ureide Test in Breath and Saliva Samples
Source: J Agric Food Chem. 2025 Apr 15;73(17):10304–15. doi: 10.1021/acs.jafc.5c00534 (PMC12046600; doi:10.1021/acs.jafc.5c00534)
Supplement: Supplementary file 1 — jf5c00534_si_001.pdf [file jf5c00534_si_001.pdf]

Measurement of oro-caecal transit time in LPS-treated pigs fed high and low fibre diets using the lactose-^{13}C-ureide test in breath and saliva samples

Mariagrazia Cavalleri[a], Quentin L. Sciascia[a], Solvig Görs[a], Andreas Vernunft[a], Henry Reyer[a], Klaus Wimmers[a,b], Jürgen Zentek[c], Jeannette Kluess[d], Sven Dänicke[d] and Cornelia C. Metges[a,b]*

[a]Research Institute for Farm Animal Biology (FBN), 18196 Dummerstorf, Germany

[b]University of Rostock, Faculty of Agricultural and Environmental Sciences, 18059 Rostock, Germany

[c]Freie Universität Berlin, Institute of Animal Nutrition, 14195 Berlin, Germany

[d]Federal Research Institute for Animal Health, Institute of Animal Nutrition, 38116 Braunschweig, Germany

*Email: metges@fbn-dummerstorf.de

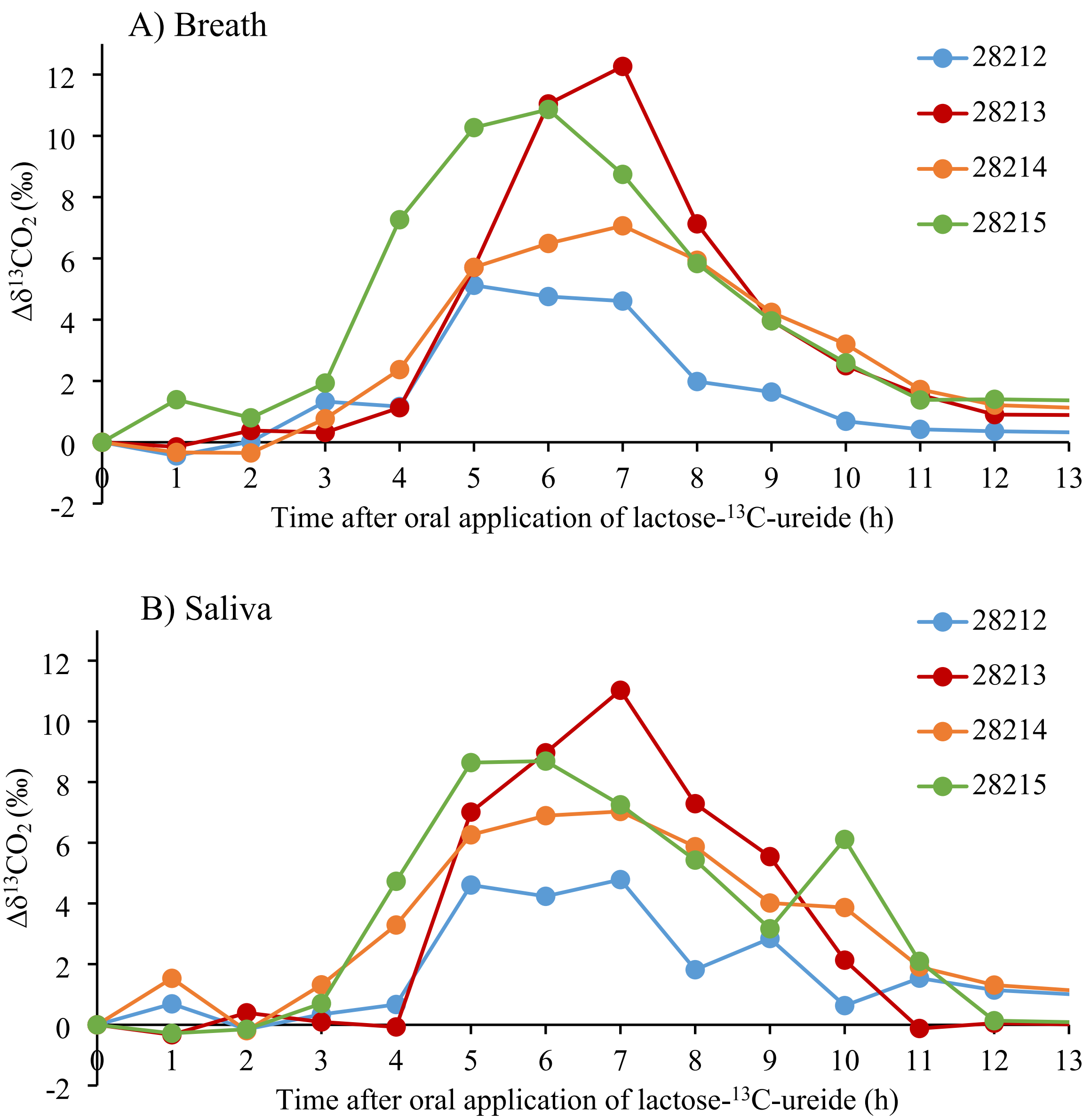

Figure S1. Pre-trial $^{13}CO_2$ enrichment curves after oral lactose-^{13}C-ureide **(**L^{13}CU) administration measured in breath (A) and saliva (B) CO_2.

Results: To achieve the fastest and highest increase in ^{13}C levels in breath and saliva, it was found that administering a single pre-dose of 400 mg 12 h before giving L^{13}CU immediately before the morning feeding was the most effective approach.

Table S1. Nutrient and energy contents of diets fed to sows during pregnancy and lactation, and the experimental piglets before (14-28 d) and after (28-35 d) weaning, and during the pre-experimental period (35-49 d)

item	brand	CP, %	EE, %	CF, %	ME, MJ/kg
sows[a]					
pregnancy		12.6	3.80	9.00	11.4
lactation	Provital	16.5	6.00	5.30	13.2
piglets					
14 - 28 d	Immuno-G[a]	18.8	9.20	2.70	15.2
28 - 35 d	Turbostart[b]	19.0	7.50	3.00	14.8
35 - 49 d	Porcistart[b]	18.0	5.30	4.00	13.8

Abbreviations: CP, crude protein; EE, ether extract; CF, crude fibre; ME, metabolisable energy.
[a]Manufacturer: UNA-HAKRA Hanseatische Kraftfuttergesellschaft mbH, Neuhöfer Damm 116, Hamburg, Germany.
[b]Manufacturer: Trede & von Pein GmbH, Dammfleth, Germany.

Table S2. Pre-trial experimental set-up for assessing dosages and sampling times for the lactose-^{13}C-ureide test in breath and saliva

		piglet ID			
age (d)	item	28212	28213	28214	28215
		mg			
d75	LU pre-dose 15h prior L^{13}CU test	200	-	200	-
	LU pre-dose 12h prior L^{13}CU test	200	400	200	400
d76	L^{13}CU dose 1h before feed	500	500	-	-
	L^{13}CU dose 5 min before feed	-	-	500	500

Abbreviations: L^{13}CU, lactose-^{13}C-ureide; LU, lactose ureide.

Table S3. Correlation coefficients (r) and *p*-values between breath and saliva $\Delta\delta^{13}C$, OCTT, E_{max}, T_{max} and AUC values of pigs fed low fibre or high fibre diets and pre-challenged with LPS or NaCl, during the lactose-^{13}C-ureide test

items	low fibre[a]				high fibre			
	LPS		NaCl		LPS		NaCl	
	r	*p*	r	*p*	r	*p*	r	*p*
$\Delta\delta^{13}C$, ‰	0.901	< 0.001	0.956	< 0.001	0.929	< 0.001	0.952	< 0.001
OCTT, h	0.488	0.183	0.919	< 0.001	0.705	0.034	0.940	< 0.001
$E_{max,}$ $\Delta\delta^{13}C$ ‰	0.901	0.001	0.825	0.003	0.942	< 0.001	0.596	0.090
$T_{max,}$ h	0.945	< 0.001	0.961	< 0.001	0.873	0.002	0.915	0.001
AUC, $\Delta\delta^{13}C$ ‰ ·h	0.563	0.114	0.835	0.003	0.970	< 0.001	0.659	0.053

Abbreviations: AUC, area under the enrichment-time curve; E_{max}, maximum enrichment; LF, low fibre; LPS, lipopolysaccharide challenge; HF, high fibre; NaCl, sodium chloride (control group); OCTT, oro-caecal transit time; r, correlation coefficient; T_{max}, time to reach the maximum enrichment; Trt, treatment (LPS challenge or NaCl control).

[a]LF-LPS/HF-NaCl n = 6-9; LF-NaCl n = 6-10; HF-LPS n = 6-8.

Table S4. Urea concentration in urine samples from pigs fed low fibre or high fibre diets and pre-challenged with LPS or NaCl, during the lactose-^{13}C-ureide test

items^{a,b}	low fibre		high fibre		*p* values^{c}		
	LPS	NaCl	LPS	NaCl	Diet	Time	Trt
urinary urea, mmol/L					0.736	< 0.001	< 0.001
-12-0 h	247 ± 25.8^{c}	109 ± 25.8^{d}	237 ± 31.3^{c}	100 ± 30.6^{d}			
0-3 h	216 ± 26.4^{ac}	74.5 ± 26.9^{d}	131 ± 30.3^{b}	87.7 ± 27.2			
3-6 h	161 ± 27.8^{b}	101 ± 26.3	284 ± 31.1^{ac}	88.5 ± 28.5^{d}			
6-9 h	213 ± 25.8^{c}	127 ± 26.5^{d}	244 ± 27.0^{c}	132 ± 27.2^{d}			
9-12 h	133 ± 25.8	63.2 ± 27.7	136 ± 27.9	73.4 ± 28.1			

Abbreviations: LPS, lipopolysaccharide challenge; NaCl, sodium chloride (control group); Trt, treatment (LPS challenge or NaCl control).

^{a}Values are least-square means ± standard error of the mean (SEM); LF-LPS, $n = 4$; LF-NaCl, HF-NaCl, HF-LPS, $n = 3$.

^{b}Values with a or b lower case letters within row differ between diets within treatment ($p < 0.05$). Values with c or d lower case letters within row differ between treatments within diet ($p < 0.05$).

^{c}*F*-test; Diet × Trt × Time ($P < 0.001$).

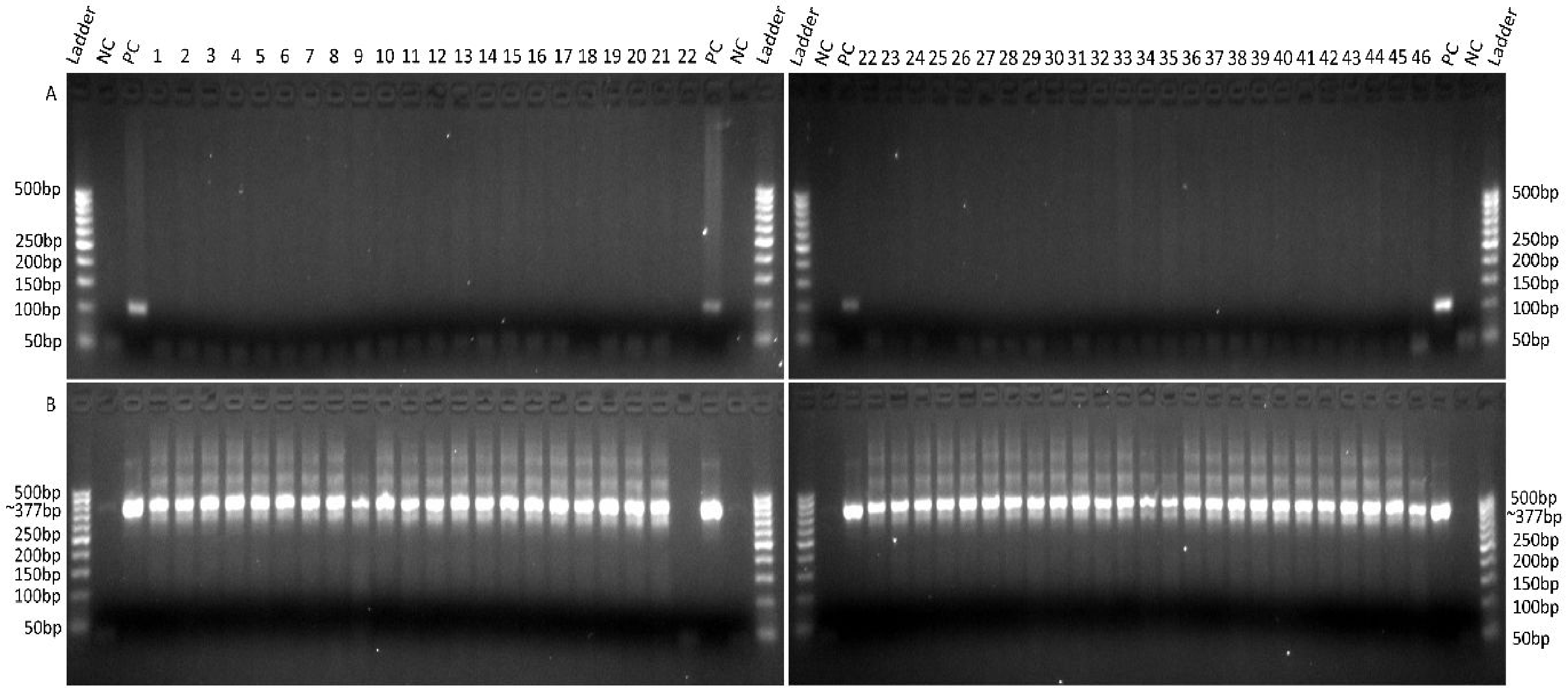

Figure S2. PCR electrophoresis gels of faecal microbial DNA sampled on the LPS-challenge day (d75). A) Specific *C. innocuum* primers. B) 16S primers. Abbreviations: HF, high fibre; LF, low fibre; LPS, lipopolysaccharide challenge; NaCl, sodium chloride (control group).

Lanes from left to right: 50 bp ladder, negative control (NC), positive control (PC), lanes 1 to 8 HF-LPS samples (respective time points relative to LPS-injection: 0, 4, 12, 0, 0, 0, 4, 12 h), lanes 9 to 22 HF-NaCl samples (0, 4, 12, 0, 4, 12, 0, 4, 0, 4, 12, 0, 4, 12 h), lanes 23 to 33 LF-LPS samples (0, 4, 12, 0, 12, 0, 4, 12, 0, 4, 12 h), lanes 34 to 46 LF-NaCl samples (0, 12, 0, 4, 12, 0, 4, 12, 0, 4, 0, 4, 12 h), PC, NC, 50 bp ladder.

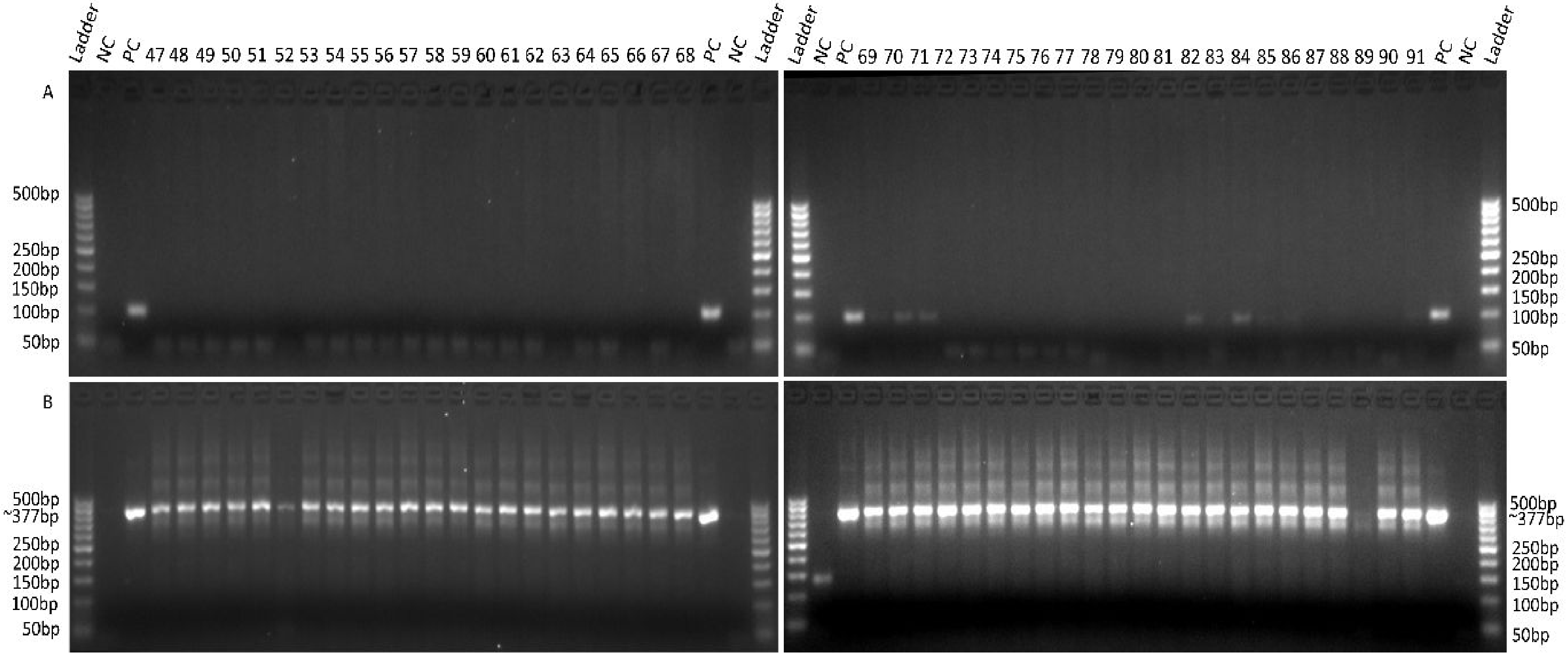

Figure S3. PCR electrophoresis gels of faecal microbial DNA sampled on the lactose-^{13}C-ureide (L^{13}CU) test day (d76). Panel A) Specific *C. innocuum* primers. Panel B) 16S primers. Abbreviations: HF, high fibre; LF, low fibre; LPS, lipopolysaccharide challenge; NaCl, sodium chloride (control group).

Lanes from left to right: 50 bp ladder, negative control (NC), positive control (PC), samples from 47 to 57 HF-LPS (respective time points relative to L^{13}CU administration: 0, 6, 12, 6, 12, 0, 6, 12, 0, 6, 12 h), 58 to 68 HF-NaCl (0, 6, 12, 0, 6, 12, 0, 6, 12, 0, 6 h), 69 to 82 LF-LPS (0, 6, 12, 6, 12, 0, 6, 12, 0, 6, 12 h), 83 to 91 LF-NaCl (6, 12, 0, 6, 12, 6, 12, 0, 6, 12, 6, 12 h), PC, NC, 50 bp ladder.
